# Clonal Hematopoiesis Analyses in Clinical, Epidemiologic, and Genetic Aging Studies to Unravel Underlying Mechanisms of Age-Related Dysfunction in Humans

**DOI:** 10.3389/fragi.2022.841796

**Published:** 2022-03-08

**Authors:** Kenneth Walsh, Nalini Raghavachari, Candace Kerr, Alexander G. Bick, Steven R. Cummings, Todd Druley, Cynthia E. Dunbar, Giulio Genovese, Margaret A. Goodell, Siddhartha Jaiswal, Jaroslaw Maciejewski, Pradeep Natarajan, Anastasia V. Shindyapina, Alan R. Shuldiner, Erik B. Van Den Akker, Jan Vijg

**Affiliations:** ^1^ University of Virginia, Charlottesville, VA, United States; ^2^ National Institute on Aging, NIH, Bethesda, MD, United States; ^3^ Vanderbilt University, Nashville, TN, United States; ^4^ University of California, San Francisco, San Francisco, CA, United States; ^5^ Angle Biosciences, St. Louis, MO, United States; ^6^ National Heart, Lung and Blood Institute, NIH, Bethesda, MD, United States; ^7^ Broad Institute, Cambridge, MA, United States; ^8^ Baylor College of Medicine, Houston, TX, United States; ^9^ Stanford University, Palo Alto, CA, United States; ^10^ Cleveland Clinic, Cleveland, OH, United States; ^11^ Massachusetts General Hospital, Boston, MA, United States; ^12^ Harvard Medical School, Boston, MA, United States; ^13^ Regeneron Pharmaceuticals, Tarry Town, NY, United States; ^14^ Leiden University, Leiden, Netherlands; ^15^ Department of Biomedical Data Sciences, Leiden University Medical Center, Leiden, Netherlands

**Keywords:** clonal hematopoiesis, aging, CHIP, somatic mutations, longevity

## Abstract

Aging is characterized by increased mortality, functional decline, and exponential increases in the incidence of diseases such as cancer, stroke, cardiovascular disease, neurological disease, respiratory disease, etc. Though the role of aging in these diseases is widely accepted and considered to be a common denominator, the underlying mechanisms are largely unknown. A significant age-related feature observed in many population cohorts is somatic mosaicism, the detectable accumulation of somatic mutations in multiple cell types and tissues, particularly those with high rates of cell turnover (e.g., skin, liver, and hematopoietic cells). Somatic mosaicism can lead to the development of cellular clones that expand with age in otherwise normal tissues. In the hematopoietic system, this phenomenon has generally been referred to as “clonal hematopoiesis of indeterminate potential” (CHIP) when it applies to a subset of clones in which mutations in driver genes of hematologic malignancies are found. Other mechanisms of clonal hematopoiesis, including large chromosomal alterations, can also give rise to clonal expansion in the absence of conventional CHIP driver gene mutations. Both types of clonal hematopoiesis (CH) have been observed in studies of animal models and humans in association with altered immune responses, increased mortality, and disease risk. Studies in murine models have found that some of these clonal events are involved in abnormal inflammatory and metabolic changes, altered DNA damage repair and epigenetic changes. Studies in long-lived individuals also show the accumulation of somatic mutations, yet at this advanced age, carriership of somatic mutations is no longer associated with an increased risk of mortality. While it remains to be elucidated what factors modify this genotype-phenotype association, i.e., compensatory germline genetics, cellular context of the mutations, protective effects to diseases at exceptional age, it points out that the exceptionally long-lived are key to understand the phenotypic consequences of CHIP mutations. Assessment of the clinical significance of somatic mutations occurring in blood cell types for age-related outcomes in human populations of varied life and health span, environmental exposures, and germline genetic risk factors will be valuable in the development of personalized strategies tailored to specific somatic mutations for healthy aging.

## Introduction

**FIGURE 1:**
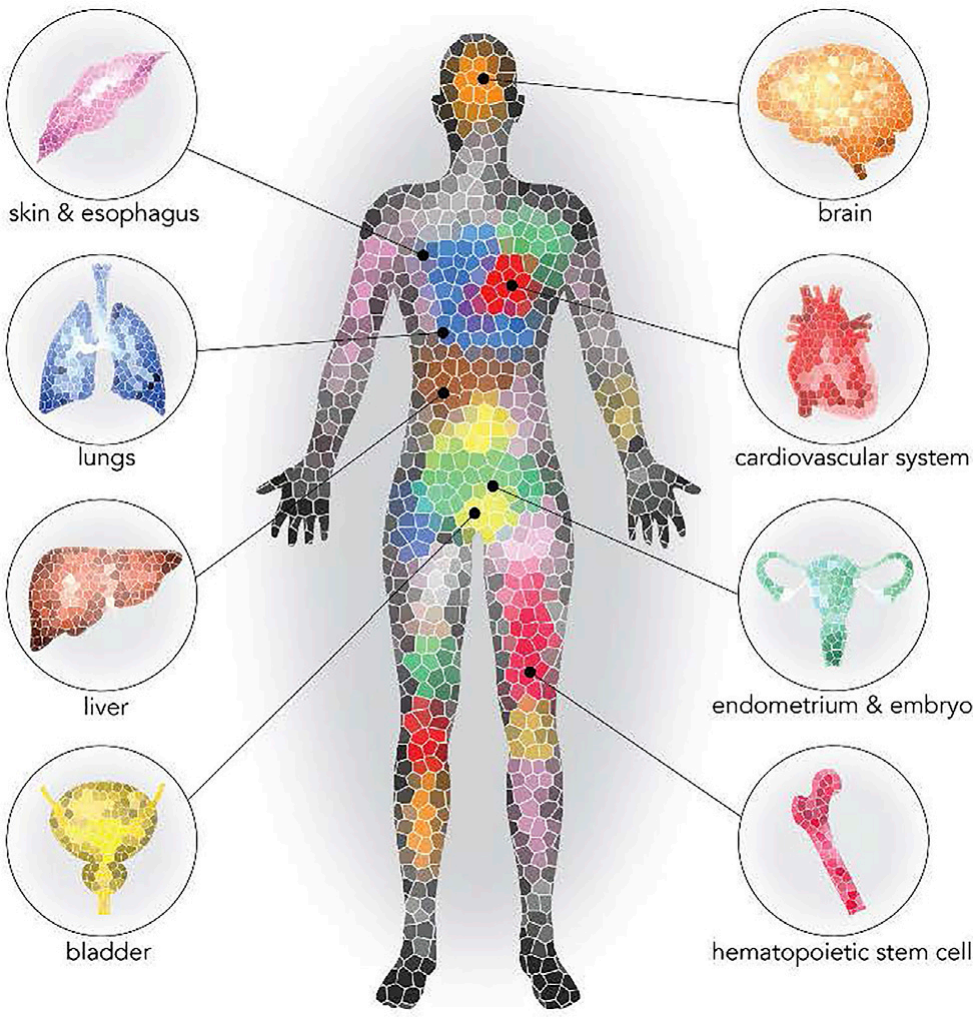
Prevalence of clonal expansion in normal tissues (Sano et al., 2020). Reprinted with permission.

Aging is characterized by widespread functional declines at the cellular and tissue levels leading to increased disease susceptibility and mortality. However, it remains unknown whether aging has a unifying causal mechanism or whether it is the result of multiple disparate mechanisms. With age, DNA accumulates somatic mutations (Fuster and Walsh, 2018; Vijg, 2021). Most of these mutations have little or no effect on normal cell physiology, but rarely a mutation can provide a selective advantage to the cell in which they occur. In the hematopoietic system, the result is a clonal expansion in the peripheral blood since the mutated hematopoietic progenitors maintain the ability to differentiate into leukocytes. The accumulation of mutations coupled with age-related changes in DNA repair and reduced regenerative potential may contribute to expansion of cellular clones within tissues (Ermolaeva and Schumacher, 2014; Gibson and Steensma, 2018; Vijg and Dong, 2020). Recent advances in DNA sequencing methodologies reveal that age-associated somatic mutation accumulation is remarkably prevalent, revealing a degree of cellular heterogeneity within tissues that was previously underappreciated. Notably, the age-associated expansion of mutant clones has been observed in multiple tissues (Figure 1). In most of these tissues, the consequences of the age-associated increases in somatic mosaicism remain to be established. However, mutant clones in the hematopoietic system could be particularly impactful on the organism as these cells migrate throughout the body and have important surveillance functions.

**FIGURE 2:**
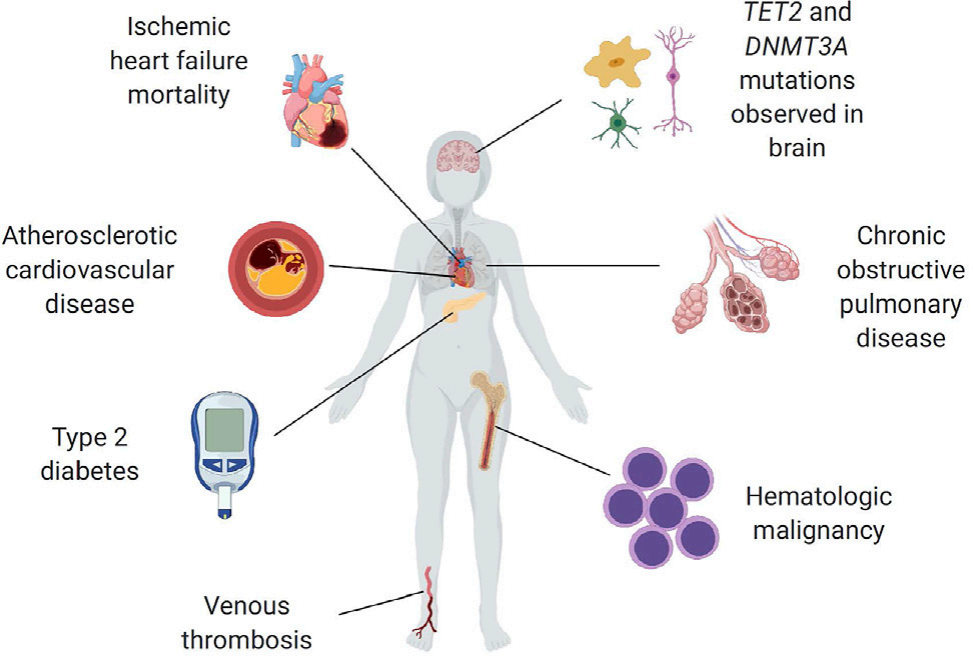
Summary of reported CHIP associations to age-related diseases (Jaiswal, 2020). Reprinted with permission.

Similar to other populations of stem cells, hematopoietic stem cells (HSCs) have an increased tendency to acquire mutations as they proliferate due to multiple physiologic and pathologic mechanisms (Goodell and Rando, 2015; Al Zouabi and Bardin, 2020). For the purposes of this discussion, we will use clonal hematopoiesis (CH) as an umbrella term to refer to the presence of an expanded mutant clone of any sort within the blood, excluding the reactive expansion of immune cells within lymphoid organs and frank malignancy (Silver et al., 2021). Based upon next generation DNA sequencing (NGS) data, approximately 15–20% of people of age 70 or older carry a detectable cancer-associated somatic mutation at ≥2% variant allele frequency within known driver genes in a substantial proportion of their blood cells. This phenomenon, known as clonal hematopoiesis of indeterminate potential (CHIP), occurs most commonly as a result of mutations in the transcriptional regulators DNMT3A, TET2 and ASXL1 (Link and Walter, 2016; Bejar, 2017; Jaiswal and Ebert, 2019; Libby et al., 2019; Mooney et al., 2021). Understandably, individuals with CHIP display modestly increased risk of developing a hematologic malignancy, although the vast majority of individuals with this condition do not develop blood cancer. Recent evidence has also associated CHIP with a variety of adverse outcomes including atherosclerotic cardiovascular disease, heart failure (HF) and venous thrombosis (Jaiswal et al., 2014; Jaiswal and Ebert, 2019; Jaiswal and Libby, 2020) (Figure 2). Studies in different experimental systems indicate that CHIP is a causal risk factor for cardio-metabolic diseases and that it can activate pro-inflammatory signaling in myeloid cells (Fuster et al., 2017; Jaiswal et al., 2017). Some of these findings have been corroborated in human studies, and they generally support the concept that CHIP reflects a new mechanism of cardiovascular disease (and other age-related diseases) that shares mechanistic features with hematologic malignancies. In addition to CHIP, CH can also develop from mosaic chromosomal alterations (CAs’ i.e., structural and copy number variants), neutral drift or other potential mechanisms. For instance, CAs in the form of gains, losses, and copy neutral loss of heterozygosity (CN-LOH) events can be readily detected at cell fractions as low as 1% from DNA microarray data, when using a statistical framework that leverages haplotype information (Loh et al., 2018; Liyen Loh et al., 2020; Po-Ru Loh et al., 2020). Moreover, CAs of somatic origin with an allele frequency threshold of >2% are virtually undetectable in blood-derived DNA of individuals below the age of 45 but are detectable in more than 20% of individuals older than 80 years. These events are often precursors to more malignant versions leading to blood cancer diagnoses, and can be associated with inherited germline variants, with both cis and trans effects.

While interest in CH/CHIP is growing rapidly, a number of unresolved issues remain, and mechanistic knowledge associated with somatic mutations in health and disease as a function of age is limited. Hence, the National Institute on Aging (NIA) convened a workshop in March 2021 involving experts from academia, industry, and government. The major goals of the workshop were to review the significance of CH/CHIP in aging phenotypes, with a particular focus on optimal sequencing and computational methodologies which could be used for the analysis of existing biospecimens from epidemiological cohorts with extensive physiologic and genetic data to assess the clinical significance of somatic mutations for a variety of age-related outcomes. During the 2-day virtual workshop comprising of four different sessions, a multidisciplinary group of experts discussed the relationship of somatic mutation derived CHIP and other CH events to age-related diseases and longevity. These discussions emphasized the importance of correlating CH/CHIP with physiological factors, environmental exposures, and germline genetic risk factors with the goal of developing a better understanding of how clonal events impact longevity and the risk of age-related disease. Technologies for the measurement of CH/CHIP and translational models for functional studies of these clonal events were also considered.

## CH/CHIP Mutations and Their Relationships to Aging Conditions

Based upon conventional NGS (without the use of error correction strategies), the prevalence of CHIP varies with age, ranging from less than 1% in those younger than 40 years to more than 15% in those 70 years and older (Papa et al., 2020). A combined analysis of DNA from leukocytes from nearly 130,000 individuals provided additional evidence for the association of mosaicism with increasing age as well as suggested elevated frequencies of mosaicism in males (Machiela et al., 2015). Work over the past decade has uncovered that CH/CHIP is a universal condition characterized by the positive selection of certain somatic mutations in hematopoietic stem cells in aging humans. Clonal expansion driven by leukemia-associated somatic mutations, such as DNMT3A, ASXL1, or TET2, are associated with an increased risk of blood cancers which is nearly tenfold compared to age-matched individuals who do not display detectable CHIP, with an absolute risk of progression is about 1% per year (Jaiswal et al., 2017).

Regarding a potential role of CHIP mutations in other conditions, emerging evidence from epidemiologic and other clinical studies suggest strong associations between CHIP and CVD. CHIP was found to be associated with an increased risk for coronary artery disease (CAD) by two-fold and premature MI by fourfold in the general population independently of age or other traditional cardiovascular risk factors (Jaiswal et al., 2017; Libby et al., 2019; Evans et al., 2020; Min et al., 2020). In a cohort of individuals with ischemic heart failure (HF), Dorsheimer and others (Dorsheimer et al., 2020) observed an association between CHIP, due to mutations in DNMT3A and TET2 genes, with a marked increase in the number of hospitalizations and risk of death from HF. These observations, though from a smaller number of patients, tend to support the involvement of CHIP not only in the pathogenesis of MI and stroke, but also in HF prognosis (Yu et al., 2021). In tandem with aging, the prevalence of comorbidity increases in patients with chronic HF and nearly half of patients with HF have five or more comorbidities associated with a pro-inflammatory state. Diabetes occurs in approximately 40% of male patients and in 30% of female patients with HF and is associated with a two-fold increased risk of developing CHIP. Individuals with both diabetes and CHIP have a higher burden of cardiovascular comorbidities than those with diabetes alone (Mooney et al., 2021). Self-reported asthma and chronic obstructive pulmonary disease were also associated with TET2-based CH, and whole-genome sequencing–based detection of CH was associated with chronic pulmonary disease (Cook et al., 2019). However, a comprehensive landscape of potential associations between CH and other comorbidities of aging requires further development. Given the important intersections among aging and vascular disease in the pathogenesis of HF and other diseases, CHIP reflects a ripe target for further assessment in this growing group of patients who currently lack evidence-based therapy. Whether CHIP status will allow personalization of therapy for these patients and others remains an open avenue for future work based on the mechanistic findings on CHIP in multiple age-related disease models.

The availability of DNA-methylation (DNAm) data from large epidemiological cohorts has advanced our understanding of the phenomenon of epigenetic aging as measured by changes in DNAm patterns that change with age influencing health and lifespan (López-Otín et al., 2013). DNAm involves the addition of a methyl group to the 5′ position on cytosines in cytosine guanine dinucleotides, referred to as CpGs and the analyses of the methylation values of tens to hundreds of CpGs in the genome allows the construction of “epigenetic clocks”. Epigenetic clocks predict age or age-related phenotypes and identify individuals whose biological age is greater (i.e., an older epigenome) than their chronological age. This condition is referred to as “age acceleration”. Age acceleration has been shown to be associated with increased risk of CHD. It was hypothesized that CHIP may be an acquired genetic factor associated with epigenetic age acceleration. Although age is the predominant factor that contributes to the development of clonal hematopoiesis, recent studies that associated clonal hematopoiesis with age acceleration through the examination of the “epigenetic clock” showed that clonal hematopoiesis was found to be associated with 3.7 and 4.5 years of accelerated age, and individuals with TET2-mediated clonal hematopoiesis displayed the greatest accelerations of epigenetic aging (6.1 and 6.4 years) compared with those individuals with no detectable clonal hematopoiesis. (Robertson et al., 2019; Nachun et al., 2021).

Analyses of Framingham Heart Study, Jackson Heart Study, and the Women’s Health Initiative together comprising over 4,000 individuals showed an association to CHIP (Nachun et al., 2021). Upon analysis, CHIP was found to be modestly associated with mortality with a hazard ratio of about 1.3 and interestingly, stratifying CHIP carriers by methylation status showed that the 60% of CHIP carriers who do not display accelerated epigenetic aging show no increased risk of mortality. In contrast, the CHIP-positive group that displays epigenetic age acceleration has a hazard ratio of nearly 3.0. Similarly, when the analysis was performed for CHD, comparing groups with and without accelerated epigenetic aging, the 10-year risk of developing CHD was higher in the group with epigenetic aging (over 20%). Importantly, individuals displaying accelerated epigenetic age along with CHIP appear to be at a higher risk (60%) for mortality after a first heart attack than those individuals with just CHIP alone or with just accelerated epigenetic age alone after a first heart attack. Consistent with a two-hit model of epigenetic aging and CHIP, either one by itself does not particularly lead to high risk for adverse outcomes, but the combination of the two markedly increases the risk for various adverse outcomes (Nachun et al., 2021).

Whole genome analyses in centenarians and other long-lived individuals in an Icelandic population (Zink et al., 2017) and exome sequencing and association study in approximately 60,000 participants in a large U.S. health care system (unpublished) revealed an increased prevalence of CHIP variants with increasing age. In populations of Ashkenazi Jewish and German descent, CHIP variants are less frequent in the offspring of long-lived parents compared to age-matched controls suggesting the presence of inherited mechanisms that prevent or delay somatic mutations in long-lived families (Shuldiner—unpublished). Similar analyses in Rotterdam study participants aged 80 years and older, and the Leiden Longevity Study participants aged 89 years and above failed to identify an association between CHIP mutations and mortality, suggesting the existence of protective mechanism(s)/factors in these subjects (van den Akker et al., 2016). As a follow up, a longitudinal analysis was performed in a female participant of the 100-plus study, who donated peripheral blood at ages 103, 110, and 111 years to investigate clonality in peripheral blood samples and sorted cell subsets (van den Akker et al., 2021). Interestingly, the investigators identified a highly dominant DNMT3A-mutated HSC clone, with a complex sub clonal architecture, and observed ongoing sub-clonal dynamics within the 9-year timeframe. A pilot study on HSC output from this individual revealed the major contribution of DNMT3A-mutated HSC to myeloid cells (78–87%) and to a small proportion of T-cells (11%) and B-cells (6–7%). Moreover, the HSC contributed to a significantly larger proportion of CD4^+^ T-cells (22%) than CD8^+^ T-cells (6%). Nevertheless, T-cells showed a robust proliferation when challenged *in vitro* as determined by T-cell receptor excision circle (TREC) analysis and were characterized by a surprisingly high TREC content indicative of an ongoing generation of naive T-cells indicating functional T-cell immunity (van den Akker et al., 2021). While this exploration is limited to only a single individual, it does suggest an independence between the two studied aspects of the aging hematopoietic system. Further studies in large cohorts are needed to determine whether further research is warranted to understand the impact of CHIP on the phenotypic and functional parameters of adaptive immunity.

## Pathophysiologic Mechanisms Contributing to Conditions—Inflammation, Cell Kinetics

Experimental studies of CH thus far, have provided some mechanistic evidence that sequence variation in a number of CHIP genes is accompanied by increased inflammation because of activation of the inflammasome complex in hematopoietic cells (Sano et al., 2018a; Sano et al., 2018b). In experimental hypercholesterolemia-prone mice engrafted with TET2−/− bone marrow (including myeloid-specific TET2 deficiency), larger atherosclerotic lesions were found when compared to those receiving control bone marrow (Jaiswal et al., 2017; Fuster et al., 2020). These myeloid cells displayed enhanced induction of inflammatory mediators such as IL6, and IL-1β. Inhibition of the NLRP3 inflammasome has been shown to mitigate atherosclerosis among hypercholesterolemic mice transplanted with the TET2−/− bone marrow (Fuster et al., 2020). More recently, these experimental studies have been expanded to other CVD models and to other driver genes, including DNMT3A, JAK2, TP53 and PPM1D (Sano et al., 2018c; Sano et al., 2019; Fidler et al., 2021; Sano and Walsh, 2021; Yura et al., 2021). Collectively, these data indicate that clonal hematopoiesis leads to the overactivation of inflammatory responses in a gene-specific manner. These studies have also identified common features between these different forms of clonal hematopoiesis. Most notably, overactivation of the IL-1β and/or IL-6 signaling pathway was observed among multiple driver genes. Based on data obtained in animal models, CHIP increased cardiovascular mortality may be due to accelerated inflammasome-mediated tissue injury and proinflammatory interactions. Studies in mice support the causality of the relationship between CHIP and increased expression of inflammatory genes in innate immune cells, potentially explaining the link between these mutations and increased CVD risk. Notably, studies in aged mice have shown that a model of clonal hematopoiesis mediated by TET2 loss-of-function in HSCs leads to accelerated age-associated insulin resistance and cardiac dysfunction (Fuster et al., 2017; Wang et al., 2020).

The experimental findings relating clonal hematopoiesis to excessive inflammation via IL-1B and/or IL-6 have been validated in a few clinical analyses utilizing the TOPMed, United Kingdom Biobank, and CANTOS cohorts (Ridker et al., 2018; Bick et al., 2020a; Bick et al., 2020b; Evans et al., 2020). While CHIP has been shown to activate pro-inflammatory cytokines, other markers of inflammation such as white blood count (WBC), neutrophil count, C-reactive protein (CRP) and erythrocyte sedimentation rate (ESR) are typically not elevated in individuals with CHIP (Jaiswal et al., 2014; Cook et al., 2019). The association between CHIP and elevated inflammatory factors is of particular interest, given that increased inflammation underlies many age-related conditions. Furthermore, the presence of a frequently occurring sequence variation of the IL-6 receptor, that results in reduced IL-6 signaling, is associated with a reduced risk for cardiovascular events in individuals who carry DNMT3A or TET2 CHIP-driver sequence variations (Bick et al., 2020b). Collectively, these data provide a compelling case that excessive inflammation links CHIP to disease processes. However, further studies are needed to develop a better understanding of CHIP impacts different age-related clinical outcomes, and better define the role of inflammation in how CHIP impacts various physiologic functions in distinct diseases.

It is widely believed that clonal hematopoiesis results from the selective advantage of variant stem cells that allows them to outcompete others so that their progeny become overrepresented. At the level of the hematopoietic stem cell, it is believed that these somatic mutations confer enhanced responses to inflammation and protection from inflammation-induced damage (Cai et al., 2018). Because these same mutations lead to systemic elevations of IL-1β and IL-6 levels, it can be speculated that a positive feedback loop is created that can lead to a selective advantage for mutant cell expansion in the bone marrow niche. In this regard, exposure of mice to the pro-inflammatory mediator, tumor necrosis factor-α (TNF-α), has been shown to promote the expansion of TET2 mutant clones and exposure to inflammatory stress in myeloid cells results in the rapid increase in frequency and absolute number of TET2-mutated myeloid cells (Abegunde et al., 2018). Thus, it has been proposed that the role of inflammation in CHIP is bidirectional creating a positive feedback loop; CHIP promotes systemic inflammation and the consequent pro-inflammatory cytokine release further promotes the clonal expansion (Mooney et al., 2021). Mutations in the DNMT3A gene represent the most prevalent driver gene detected in individuals with CHIP. To illustrate the selective advantage associated with this driver gene, studies were performed with transplanted wild-type or DNMT3A-knockout hematopoietic stem cells. Their studies showed that the DNMT3A-knockout stem cells generated a greater proportion of hematopoietic stem cells after the transplantation with a large bias toward self-renewal (Challen and Goodell, 2020).

The rate of development of overt neoplasia in patients with CHIP as currently defined, is 0.5–1% per year (Steensma, 2018). In relation to this, DNMT3A R882 mutations were shown to act as dominant negatives when heterodimerizing with a wild-type protein. Mutations elsewhere in the gene are mostly incapable of dimerizing the wild type protein, and are thus believed to be functionally inert. (Russler-Germain et al., 2014). While the DNMT3A mutations at R882 hotspot dominates in acute myeloid leukemia (AML), other mutations spread throughout the gene are collectively more common in CH suggesting that differences in the potency of the mutant isoform may mechanistically contribute to different diseases, indicating a prognostic utility for patients (Yang et al., 2015). Contributing factors to cancer progression, other than acquisition of secondary mutations in hematopoietic cells (i.e., stronger leukemia drivers), are incompletely understood. Potential contributors to clonal progression include disordered endogenous immunity in the context of increased proliferative pressure, telomere shortening leading to chromosomal instability, an unhealthy marrow microenvironment that favors expansion of clonal stem cells. Acquisition of new mutations while failing to support healthy hematopoiesis, aging associated changes in hematopoietic stem cells including an altered DNA damage response, an altered transcriptional program, and consequences of epigenetic alterations could contribute to clonal progression. With regard to CVD, the clinical management of patients with CHIP may include monitoring for hematological changes and reduction of modifiable cardiovascular risk factors. Eventually, it may include anti-inflammatory therapies and targeted approaches to prune emergent dangerous clones. Genes commonly mutated in CHIP are associated with DNA methylation, inflammation, generation of reactive oxygen species, and the DNA damage response. Factors associated with CHIP, including inflammation, CVD, metabolic disorders, and stroke are also associated with Alzheimer’s disease and other dementias. However, the association between CHIP and dementia remains unknown and warrants further investigation.

## Methodologic Challenges in Identifying CH and its Relationships to Clinical Outcomes

**FIGURE 3:**
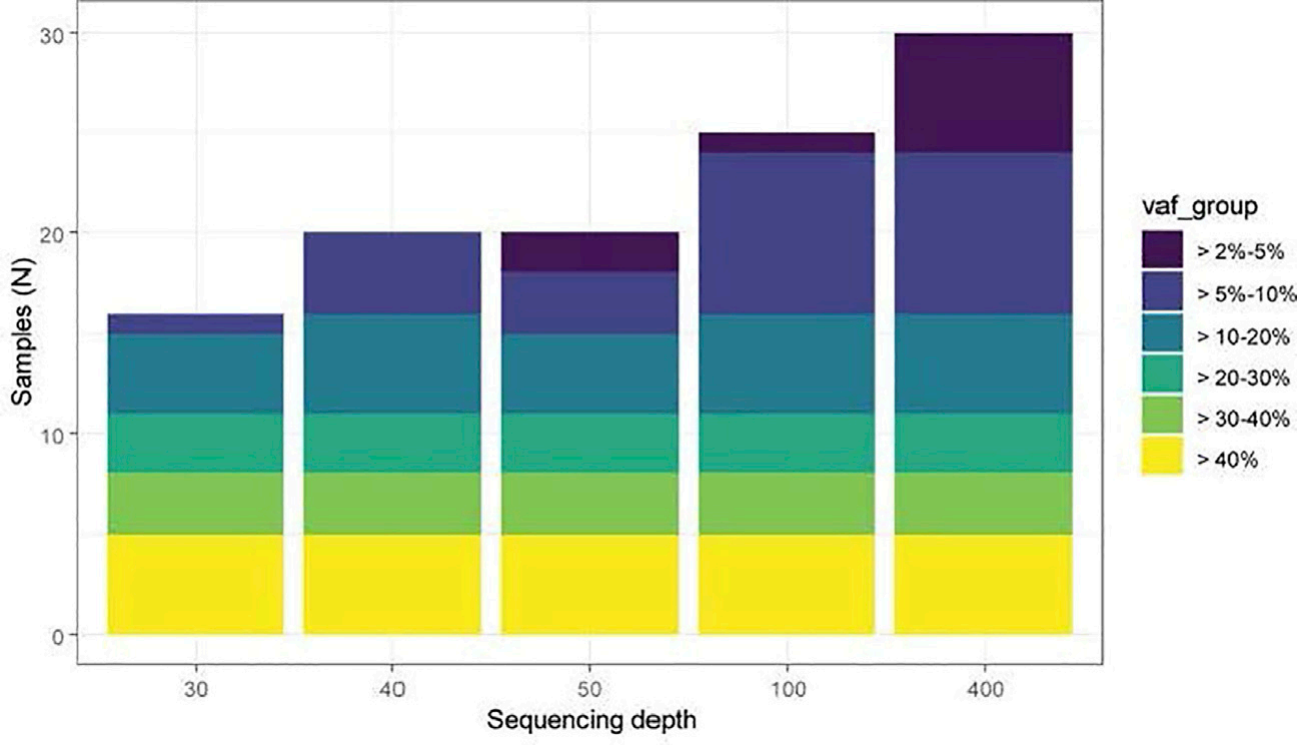
Sensitivity of CHIP detection at different sequencing depths (Bick et al., 2020a). A set of 30 samples from a previously published CHIP cohort were computationally down sampled to 30x, 40x, 50x, 100x and 400x sequencing depth. TOPMed WGS data was typically in the 40x depth range across CHIP genes. WGS data has excellent sensitivity to detect CHIP clones with VAF >10%, and ∼50% sensitivity to detect CHIP VAF 5–10%, with minimal ability to detect CHIP clones <5%.

Although somatic mutation theory of aging was hypothesized many decades ago (Failla, 1958; Szilard, 1959; Curtis and Crowley, 1963; Curtis, 1965), the lack of technologies to quantitatively measure somatic mutations in cells hindered the ability to advance this theory. With the current developments in technologies for sequencing the genome at high resolution and in a high throughput manner, the role of CH/CHIP in biological aging has gained momentum. However, diagnosing, surveilling, and understanding the biological consequences of CH continue to pose critical challenges for both basic scientists and clinicians due to lack of standards in measuring CH and questions about clinically relevant clone size. NGS with ultra-high throughput, scalability, and speed, enables researchers to survey a large number of samples and allow whole genome sequencing (WGS), exome and RNAseq at 10x to 100X coverage from small number of cells (including single cells). Figure 3 illustrates the sensitivity of mutation detection across different sequencing depths. At 30X depth of coverage, clones that are present at more than 10 or 20 percent in blood can be detected. Sequencing depth has a great impact on the detection of CHIP clones and relies on the processing of sequence data, e.g., the genomic assembly completeness and accuracy of a *de novo* assembly, the number of detected genes and expression levels in RNA-Seq, the proportion of rare variants and SNVs detected, and the accuracy of SNP calling and genotyping in whole-genome sequencing. Although the NGS platforms provide vast quantities of data, the associated error rates (∼0.1–15%) are higher and the read lengths generally shorter (35–700 bp for short-read approaches) than those of traditional Sanger sequencing platforms, requiring careful examination of the results, particularly for variant discovery and clinical applications. The observation that a small percentage of otherwise healthy adults acquire leukemia-associated CHIP mutations as a function of age was striking but based on sequencing technology with relatively poor resolution for mutations below an allele frequency of ∼5% (Jaiswal et al., 2014; Young et al., 2019). Furthermore, the physiologic and clinical consequences of a given CHIP mutation could differ depending on the blood cell subpopulation in which it occurs, but this has been little explored to date. Studies on the potential differential effects of CH in different blood cell subpopulations such as myeloid, T, and B cells could provide more insights on disease progression and the aging process. Furthermore, single-cell whole genome sequencing could provide additional insights about the possible pathogenic mechanisms of somatic mutations and genome mosaicism in tissues. Methodologies currently being optimized, such as multiple displacement amplification (MDA) in single cell analysis, could lessen the biases from error-prone amplification approaches that were used in the past and found to be allelically-biased and enriched for the mutation (Dong et al., 2017; Vijg et al., 2017; Vijg, 2021). However, MDA methods need to use a modified polymerase with proofreading capability since historically used phi29 polymerase does have the proofreading capability, resulting in a 20-fold increase in errors during amplification.

## Technologies for Sequencing Clinical Specimens and Identifying Chromosomal Alterations

Current advancements in DNA sequencing and related technologies provide a variety of options to assess various types of CH/CHIPS in clinical specimens with the potential to further investigate the functions of somatic mutations in the aging process. In an effort to overcome the sequencing errors that commonly occur with NGS, especially during the amplification process, an error-corrected sequencing (ECS) protocol has been developed to computationally eliminate sequencing errors and provide validated resolution for mutations with VAF of 0.01%, more than 100- fold less than the functional definition of CHIP (i.e., VAF of 2%) (Wong et al., 2018). ECS involves tagging individual DNA molecules with adapters containing 16 bp random oligonucleotide molecular identifiers to mitigate systematic errors in DNA sequencing. This process of affixing a unique molecular index (UMI) to each DNA fragment during target-specific amplification allows for errors to be computationally discarded and very rare mutations to be detected in heterogeneous nucleic acid samples. Applying this strategy, Druley and other researchers have demonstrated that, by age 50, nearly 100% of healthy adults harbor a complex set of clonal hematopoietic driver mutations (Young et al., 2016). While clones at these relatively young ages tend to be small, and their clinical consequences are unclear, these studies reveal the spectrum of CHIP throughout the human life span.

In addition to the commonly practiced analysis of DNA from leukocytes, analyses of cell-free DNA (cfDNA) in the blood, released by apoptosis, necrosis, and potentially other mechanisms, as an analyte for somatic mutations has attracted recent interest. Grail Inc. has developed a technology for mapping the prevalence of CH in cfDNA which was successfully applied to a cohort of participants from the Circulating Cell-free Genome Atlas (CCGA) study (Bredno et al., 2021; Chen et al., 2021). In this survey, blood was prospectively analyzed in 749 participants with no cancer at recruitment and 878 pts with newly diagnosed untreated cancer. Both white blood cells (WBC) and cfDNA were isolated and paired WBC and cfDNA targeted sequencing (507 genes, 60,000X median coverage) was performed to identify somatic variants. Unique molecular barcodes and a machine learning-based noise model was employed to achieve a specificity of one false positive variant call per Mb of genome at a limit of detection of ∼0.1% VAF. For each cfDNA variant, the study team inferred the potential clonal hematopoiesis origin from the matched participants’ WBC sample. The Grail assay was reported to be sensitive and tissue specific, and it applied this method of deep sequencing to address questions of basic biology. These studies revealed that there may not be a requirement of broad, deep sequencing for predicting disease risks. These assays might also allow the use of plasma on archived samples from epidemiological studies on aging to study the risk of frailty, disability, dementia, and other aging phenotypes.

The computational tool MOsaic CHromosomal Alterations (MoChA) (Loh et al., 2018; Po-Ru Loh et al., 2020), has been described. This technology could be applied to large human studies to detect mosaic chromosomal alterations at cell fractions as low as 1% through the detection of small imbalances between maternal and paternal haplotypes. Detection using this software could also be combined with single cell data to identify mutated cells. Applying this method to analyze data from the United Kingdom Biobank led to the detection of thousands of mosaic autosomal alterations of somatic origin (Po-Ru Loh et al., 2020). These mutations were virtually undetectable in blood-derived DNA of individuals below the age of 45 but could be identified in more than 20% of individuals older than 80 years of age.

Though there have been significant advancements in methodologies for CH analyses, challenges remain including the development of a consensus on the measurement of clonal events within the hematopoietic system, defining a clinically relevant clone size, developing a better understandings of the molecular events that contribute to clone growth, the need for longitudinal assessment of clone growth in clinical studies, and a better understanding of the clinical outcomes that result from different mutant clones.

## Factors Influencing Risk and Progression of CH and What we Need to Learn About Them

The contribution of germline genetic factors to acquisition and expansion of somatic mutations and CH risk is an emerging field that could provide deeper understanding of the underlying causes of CHIP and the interrelationships of germline and somatic mutations. In this context, NHLBI TOPMed program examined whole genome sequence data on 97,691 participants of diverse ancestries and identified 4,229 individuals with the prevalence of CHIP (Bick et al., 2020a). Identified CHIP genes were found to be associated with blood cell, lipid, and inflammatory traits. Association of a genome-wide set of germline genetic variants identified five genetic loci associated with CHIP status, including one locus at TET2 that was African ancestry specific. An in-silico-informed *in-vitro* evaluation of the TET2 germline locus identified a causal variant that disrupts a TET2 distal enhancer leading to increased hematopoietic stem cell proliferation. These findings demonstrate that the germline genetic variation can alter the effects of CHIP on hematopoietic stem cell function. Recent work has also identified an inherited polymorphism in the IL-6 receptor that reduces the likelihood of CVD in individuals with CHIP (Bick et al., 2020a; Bick et al., 2020b) While the impact of germline alterations on the frequency and impact of CHIP appear to be relatively small, the full extent to which germline factors mitigate or contribute to disease manifestations in individuals with CHIP requires further exploration. Analyses on diverse multi-ethnic cohorts could help in developing a better understanding of the genetic underpinnings that influence the occurrence of CHIP and CHIP-related outcomes.

## Clinical Applications of CH/CHIP Analyses—What Needs to be Learned?

The broad dissemination of DNA analysis capability has increased recognition of CH in various clinical settings. In hematologically normal individuals, somatic mutations may occur at an increasing frequency with age in genes that are also commonly mutated in overt myeloid malignancies such as AML and MDS (e.g., DNMT3A, TET2, and ASXL1) (Karner et al., 2019). CHIP is initiated by a spectrum of somatic mutations constituting the prototypic leukemia-initiating lesions which serve as ancestral events for CHIP-derived myelodysplastic syndrome (MDS) and secondary acute myelogenous leukemia (AML). Since CHIP-initiating lesions have a low “driver” potential *per se*, the prognostic value of their presence is confounded by a slow progression to development of a myeloid neoplasia (Palomo et al., 2020). To date, most estimates of MDS/AML risk conveyed by CHIP are unlikely to be precise as they rely on cumulative frequencies of somatic mutations. While most calculations are based on only a few serially recorded cases, stringent analysis should be based on the confirmation that the CHIP-associated mutation was also present in the corresponding myeloid neoplasm. In addition, it is possible that the presence of CHIP reflects a general risk not only for CHIP-derived, but also for other myeloid neoplasms (*de novo* vs. CHIP-derived MDS) or other cancers. Consequently, the prognostic relevance of CHIP may be multifactorial and thus much greater than that attributed to the increased risk of CHIP derived neoplasms. In addition, the type of the initiating lesion (e.g., TET2, DNMT3A, JAK2 mutations, etc.), clonal burden, and the clinical phenotype of CHIP–derived neoplasms may further confound the prognostic assessment. However, an understanding of these complex relationships may be essential for disease diagnosis, prevention, and development of therapeutic strategies. While the associations between CHIP and CVD have been well-established in experimental studies, this information has not yet entered clinical practice. There are three major roadblocks to translating this evidence to patient care: 1) lack of CHIP cohorts for most clinical trials, 2) lack of biomarkers for improving risk discrimination in CHIP, and 3) lack of mechanistic understanding of the factors underlying clonal expansion and risk of disease.

## Relationship of Modifiable Risk Factors to Development of CH Mutations and Rate of Expansion of CH Clones With Age

Smoking has been identified as a CHIP risk factor, particularly clonal events due to mutations in ASXL1 (Dawoud et al., 2020) but the factors mediating this relationship, and the relationships of other modifiable factors to the rate of increase with age for mutation-based CH have not been extensively explored. There is increasing attention to the possible benefits of screening for CHIP in asymptomatic persons and what interventions might be considered if it is found. At present, however, there is insufficient evidence to justify this, particularly a lack of clinical trial data on the benefits and harms of such screening. Given current data, therapies targeting the mutant clones, or the increased inflammatory mediators might be particularly useful for ameliorating the risk of CVD. However, the full implications of CHIP in hematology and cardiology remain to be elucidated. A growing body of research demonstrating the association between CHIP and increased risks of hematologic cancer and cardiovascular disease supports further investigation into mechanisms, causality, and novel treatment strategies to improve clinical outcomes. The use of CHIP screening may become a valuable tool for oncologists and cardiologists alike if it allows for earlier diagnosis of these conditions. Currently, there is no consensus regarding which patients should be evaluated for CHIP. Currently, it is most often identified with NGS of blood or bone marrow for the purpose of evaluating hematologic disorders, cardiovascular events in research studies, or to screen potential donors for hematopoietic cell transplantation. Additionally, the application of CHIP screening in other diseases such as dementia and other broad phenotypic cohorts could also be valuable based on preliminary findings from Women’s Health Initiative Memory Study (Hayden et al., 2020).

## Translational Animal Models for Functional Studies on CH

Epidemiological and clinical studies that identified the association between clonal expansion and diseases (CVD/Cancer) have not been able to clarify underlying mechanisms for the increased risk of diseases in carriers of CH/CHIP. For this reason, animal models have been used to investigate whether, and through which mechanisms, clones carrying mutations influence the disease process and have revealed some of the mechanisms by which CH promote CVD. For example, a mouse model transplanted with 10% knockout bone marrow cells from TET2 deficient mice developed atherosclerotic cardiovascular disease with lesions in major vessels that can lead to myocardial infarction and stroke (Fuster et al., 2017). In this study, TET2 disruption was found to promote IL 1ß and its downstream cytokine IL six through an HDAC-dependent mechanism. Animal model studies thus provide opportunities for studying mechanisms underlying clonal expansion and open the door for the development of possible therapeutic options to reduce CVD risk in CHIP carriers. In this context, there has been promising progress in the development of CHIP animal models as described below:

Like humans, mice spontaneously develop cancer with age, with standard laboratory strains predominantly dying from hematological malignancies at old age. Contrary to the general belief that mice do not develop CH, it has recently been reported that aged healthy mice acquire clonal hematopoiesis mutations at a low level (Chin et al., 2021). It has also been shown that mouse immune cells acquire somatic mutations with age (Ma et al., 2018), with some individual somatic clones of B cells to represent over half of the total B cell population. Mouse clonal B cells can expand and carry somatic mutations in genes often mutated in human lymphoma and CHIP, e.g., Trp53, Pim1, Tet2, Asxl1, and Dnmt3a. Integrating mice CH and multiomics data such as RNAseq, proteome, and DNA methylation revealed a large enrichment for cMyc targets, a critical human oncogene that is overexpressed in half of the human cancers (Shindyapina et al., 2021). The DNA methylome of clonal B cells suggests a role of epigenetic regulation in clonal selection that is similar to CHIP in humans. Accumulation of clonal B cells was associated with poorer survival of mice and age-related myeloid bias. Analysis of B cells from young animals, regular sized B cells from old animals and enlarged B cells from old animals indicated aged B cells to contain large, expanded clones, with single clones occupying on average 38% of a sample with decreased Ig diversity in the expanded B cell clones (Shindyapina et al., 2021). Interestingly, the most prevalent clones did not carry somatic mutations within CDR3 regions indicating that aged clonal B cells have not undergone somatic hypermutation in germinal centers. These findings indicate that aged mouse B cells are clonally expanded, and this is accompanied by an increase in B cell size. Collectively, there appears to be many similarities between age-related clonal expansion in mice and humans, including somatic mutations in epigenetic regulators, aberrant DNA methylation, expansions of B cells after VDJ recombination, and increased mortality. Thus, it appears that aged mice are convenient models to characterize mechanisms of age-related clonal expansion and can be employed for translational CH/CHIP studies.

Although mouse models are attractive in terms of cost, feasibility, and the ability to perform mechanistic studies by various genome manipulation approaches, there are many known differences between hematopoietic stem or progenitor cells in mice versus humans. Additionally, mouse models can be less ideal due to their short life spans, inbred nature, and a caged environment that is generally free of pathogens. It has also been reported that engineered mutations can behave differently in mice, with some of the CHIP-type mutations resulting in a rapid transition to myeloproliferative disorders or other phenotypes not observed in humans. In this context, Rhesus Macaques, with prolonged life spans in captivity of 25–40 years, outbred nature, and less restrictive environment, could be considered a more appropriate model to perform functional studies on CH/CHIP in relation to aging. Non-human primates have a high similarity in hematopoietic stem and progenitor cell (HSPC) properties to humans. Screening a cohort of naturally aged macaques for somatic CHIP mutations identified predicted loss of function (LOF) mutations in 14/53 (27%) to date, with the most frequent mutations in genes most common in human CHIP (Espinoza et al., 2019; Fan et al., 2020). A macaque model has been developed utilizing CRISPR/Cas9 technology to modify HSPCs from young adult macaques by delivering gRNAs targeting the three most frequent human CHIP genes along with Cas9 protein into HSPCs from three young adult macaques. Up to 3 years of long-term follow-up revealed reproducible and significant expansion of multiple HSPC clones with heterozygous TET2 LOF mutations, compared to the limited expansion of clones carrying DNMT3A and ASXL1 mutations, reaching a VAF of as high as almost 25%. Although there were differences in population doubling rates for mutant alleles between individuals, the three macaques shared the general pattern of a gradual but dramatic expansion of TET2-mutated clones, with most of the expanding indels resulting in predicted LOF frameshifts. These data suggest a single mutation in TET2 is sufficient for clonal expansion, and that other intrinsic and/or extrinsic factors can regulate the pace of TET2 clonal expansion. Bone marrow from these macaques exhibited hypercellularity and myeloid-predominant skewing without dysplastic changes compared with macaques of similar age previously transplanted with HSPCs edited at non-CHIP loci. Furthermore, RNA-seq indicated that TET2-disrupted myeloid colony-forming units (CFUs) and mature cells exhibited a distinct hyperinflammatory gene expression profile. The study team hypothesized that interrupting the vicious cycle of clonal expansion driven by (and driving) inflammation could halt the expansion of TET2-mutated clones and tested one animal with tocilizumab, an antibody blocking IL-6 signaling, starting 13 months after transplantation, and continuing for 4 months. The TET2 mutated allele frequency in granulocytes declined by 30% by the end of the treatment and began to increase again after withdrawal, suggesting that interruption of the IL-6 axis removes the selective advantage of mutant HSPCs. Thus, the CRISPR/Cas9-engineered rhesus macaque ARCH model recapitulates human CHIP and uncovers the impact of TET2 LOF on hematopoiesis and inflammation, as well as suggests a suppressive effect of IL-6 axis blockade in TET2-mutant clonal expansions. This robust non-human model appears to be a promising resource to examine the pathophysiology of CH/CHIP mutations and for the development of novel therapeutic interventions to maintain cellular functions. Nevertheless, high costs and difficulties in non-human primate availability act as limitations for developing these models for CH/CHIP studies.

### Perspectives and Future Directions

In summary, the workshop discussions helped to identify critical aspects of CH/CHIP mutations, aging and human health that remain largely unexplored. Examples of future research directions to advance our understanding of the clinical significance of CH/CHIP mutations and ultimately contribute to development of novel strategies to reduce disease risk in CHIP carriers include:

1) Whereas, there is ample evidence linking CHIP to mortality and increased disease risk, some forms of CHIP may be neutral or even beneficial in some disease contexts. The deleterious or protective effect of CHIP mutations as a function of age remains to be established and further studies are warranted to determine if CHIP could potentially be a maladaptive attempt to correct aging related decline in the stem cell compartment. Studies are required to determine the contributing factors affecting somatic mutation risk and identify modifiable factors that could influence the rates of CH/CHIP progression with age. In particular, longitudinal studies of CH/CHIP progression and analysis of diverse population could identify these modifiable factors.
2) High-throughput sequencing techniques and computational approaches should be developed to better analyze CH/CHIP in study populations. Related to this, methodology to detect small clones in a cost-effective manner is warranted. There appears to be need for developing a consensus on the measurement of clonal events and identifying clinically relevant clone size(s) that drive the pathophysiology.
3) Investigations are needed to elucidate the relationships of different CH/CHIP mutations to associated clinical phenomena. It is reasonable to speculate that clones originating from different driver gene mutations (or mCAs) will differentially affect disease processes. The evaluation of specific CH/CHIP mutations in middle aged and older individuals could identify those at increased risk for specific age-related diseases. Conversely, analysis of CH/CHIP in exceptionally long-lived individuals could provide information about underlying protective mechanisms that are shared by this group.
4) The examination of the differential effects of CH/CHIP in the various blood cell subpopulations is warranted and could reveal that the clinical consequences of a given CHIP mutation differs depending upon the cell subpopulation in which it occurs. This area of research has received relatively little attention and could be an important feature in understanding how CH/CHIP impacts the immune system and the aging process.
5) Studies should further elucidate the relationship between CH/CHIP somatic mutations and human germline variants, particularly those associated with aging phenotypes.
6) An important goal is to improve the evidentiary basis for health care options for persons with CH/CHIP. At present there is insufficient evidence to justify the screening of CH/CHIP in asymptomatic individuals, and there is a lack of clinical data on the benefits or harms of such screening.
7) There is a need for the development of additional animal models to understand biological mechanisms by which CH/CHIP impacts age-related disease processes. Experimental studies could also better define the impact that CH/CHIP has on the degree of biological aging. These models could potentially unravel mechanisms and functional outcomes of different driver gene clones, and ultimately identify targets for translation that could lead to therapeutic interventions for healthy aging.

The analyses of CH/CHIP in ongoing human observational and longevity studies are warranted. These studies can be augmented by comparative biology projects comprising multiple non-human species that use integrative multi-omic data analysis on multiple tissues and cell types to understand the functional outcome of the mutations. Importantly, stored biospecimens from aging studies can provide a rich resource to understand the health effects of CH/CHIP that could lead to the identification of potential interventions to mitigate the pathogenic potential of mutated clones. Importantly, implementing CH/CHIP analyses in clinical trials could identify the relationship of clonal events to outcomes in clinical drug trials, particularly those that are testing modulations of the immune system. In some cases, such analyses might indicate that the presence of CHIP influences treatment efficacy or safety sufficiently to warrant assessment of CH/CHIP status in individuals who are candidates for a given therapy.
